# A Chairman on the Cave Committee

**Published:** 1921-03-19

**Authors:** 


### A Chairman on the Cave Committee.

The National Relief Fund Criticised.

Mr. W. F. H. Thomson, chairman of the House Committee of the York County Hospital, speaking at the recent meeting of the York Workpeople's Hospital Fund Committee, said the committee under the chairmanship of Lord Cave, which was considering the future resources of voluntary hospitals, was an extremely able and fair body. He was glad to say that committee was not hidebound by the idea that there must be rate-aid or State-aid for hospitals. He was also glad that the exuberance and extravagant activity of the Ministry of Health had received a check which he hoped would restrain any effort to put voluntarily managed institutions like hospitals under the bureaucratic control of that or any other Government Ministry. York, he said, had a sore grievance because the County Hospital had not received a grant from the National Relief Fund. In York during the war they raised £12,000 by special appeals for the hospital, to maintain its financial position and provide for military needs, whereas those hospitals which made no special effort in that direction, even in towns where much wealth was created in consequence of the war, received grants from the fund.

To penalise York for having improved their position during the war was not "cricket," nor was it a business-like action. It did not encourage them to admire the way the fund was distributed. Moreover, the fixing of the date to qualify for the grant was arbitrary and did not commend itself to business men when estimating the effects of the war. York realised that now when they were faced with a large debt and could not reasonably call on the citizens for another special effort.

